# A guide to selecting high-performing antibodies for GCase (UniProt ID: P04062) for use in western blot, immunoprecipitation, and immunofluorescence

**DOI:** 10.12688/f1000research.174230.1

**Published:** 2025-12-15

**Authors:** Donovan Worrall, Charles Alende, Maryam Fothouhi, Vera Ruíz Moleón, Sara González Bolívar, Riham Ayoubi, Vincent Francis, Carl Laflamme, Peter S. McPherson

**Affiliations:** 1 Neuroscience and Neurosurgery, Montreal Neurological Institute-Hospital, Montreal, Québec, Canada

**Keywords:** P04062, GBA1, GCase, antibody characterization, antibody validation, western blot, immunoprecipitation, immunofluorescence

## Abstract

The human *GBA1* gene encodes glucocerebrosidase (GCase), a lysosomal enzyme that hydrolyzes glucosylceramides. Variants in *GBA1* and reduced GCase activity have been linked to Parkinson’s disease and Gaucher’s disease. Here we have characterized twenty-four GCase commercial antibodies for western blot, immunoprecipitation, and immunofluorescence using a standardized experimental protocol based on comparing read-outs in knockout cell lines and isogenic parental controls. These studies are part of a larger, collaborative initiative seeking to address antibody reproducibility issues by characterizing commercially available antibodies for human proteins and publishing the results openly as a resource for the scientific community. While the use of antibodies and protocols vary between laboratories, we encourage readers to use this report as a guide to select the most appropriate antibodies for their specific needs.

## Introduction

Variants in *GBA1*, which encodes the lysosomal hydrolase glucocerebrosidase (GCase), represent one of the most common genetic risk factors for Parkinson’s disease. ^1^ Biallelic mutations in *GBA1* are also causative of Gaucher’s disease, a lysosomal storage disorder. ^2^ GCase is responsible for the hydrolysis of glucosylceramide, and mutations in *GBA1* can impair the enzyme’s structure, leading to reduced activity, stability, and protein levels. ^1^ The scientific community would benefit from specific and renewable GCase antibodies to investigate how disease-related mutations affect the protein’s biochemical and cellular characteristics.

This research is part of a broader collaborative initiative in which academics, funders and commercial antibody manufacturers are working together to address antibody reproducibility issues by characterizing commercial antibodies for human proteins using standardized protocols, and openly sharing the data. ^3^ It consists of identifying human cell lines with adequate target protein expression and the development/contribution of equivalent knockout (KO) cell lines, followed by antibody characterization procedures using most commercially available renewable antibodies against the corresponding protein. ^3^ Here we characterized twenty-four commercial GCase antibodies, selected and donated by participant antibody manufacturers, for use in western blot, immunoprecipitation, and immunofluorescence (also referred to as immunocytochemistry), enabling biochemical and cellular assessment of GCase properties and function.

The authors do not engage in result analysis or offer explicit antibody recommendations. Our primary aim is to deliver top-tier data to the scientific community, grounded in Open Science principles. This empowers experts to interpret the characterization data independently, enabling them to make informed choices regarding the most suitable antibodies for their specific experimental needs. Guidelines on how to interpret antibody characterization data found in this study are featured on the YCharOS gateway ^4^ and in Table 4 of this data note. ^3^

**Table 1.** Summary of the cell lines used.

Institution	Catalog number	RRID (Cellosaurus)	Cell line	Genotype
Horizon Discovery	C631	CVCL_Y019	HAP1	WT
Horizon Discovery	HZGHC004541c001	CVCL_SP63	HAP1	*GBA1* KO
Abcam	ab255448	CVCL_0030	HeLa	WT
Abcam	ab265038	CVCL_B1SQ	HeLa	*GBA1* KO
ATCC	HTB-14	CVCL_0022	U-87 MG	WT
ATCC	CRL-4000	CVCL_4388	RPE-1	WT
Academic partner	-	-	RPE-1	*GBA1* KO

**Table 2.** Summary of the GCase antibodies tested.

Company	Catalog number	Lot number	RRID (Antibody registry)	Clonality	Clone ID	Host	Concentration (μg/μL)	Vendors recommended applications
Abcam	ab125065 **	1003589-3	AB_10973982	recombinant mono	EPR5142	rabbit	0.10	Wb
Abcam	ab128879 **	1048619-1	AB_11144121	recombinant mono	EPR5143(3)	rabbit	0.23	Wb
Abcam	ab309228 **	1089805-5	AB_3105954	recombinant mono	EPR26755-29	rabbit	1.02	other
Abcam	ab309229 **	1089807-5	AB_3105953	recombinant mono	EPR26755-42	rabbit	1.02	other
Abcam	ab55080 *	1035295-1	AB_2109076	monoclonal	200	mouse	1.00	Wb, IF
-	2020A4849 *	20231009	AB_3696746	monoclonal	1/17 (MJFF request)	mouse	1.00	NA
-	P1AM1914-001-28 *	20.02.2024	AB_3696747	monoclonal	1/23 (MJFF request)	mouse	1.00	NA
ABCD Antibodies	ABCD_AR148 **	11/15/2024	AB_3076343	recombinant mono	2D4	rabbit	0.05	NA
ABCD Antibodies	ABCD_AR150 **	11/15/2024	AB_3076344	recombinant mono	2L18	rabbit	0.02	NA
ABclonal	A19057 **	4000000500	AB_2862550	recombinant mono	ARC0500	rabbit	0.51	Wb
Bio-Techne (Novus Biologicals)	NBP2-66871 **	HO0913	NA	recombinant mono	JM10-76	rabbit	1.00	Wb, IF
Bio-Techne (R&D Systems)	MAB7410 *	CHEF0422011	AB_2938856	monoclonal	812201	mouse	0.50	Wb, IF
Cell Signaling Technology	88162 **	1	NA	recombinant mono	E2R1L	rabbit	0.08	Wb
GeneTex	GTX101267	43817	AB_1950346	polyclonal	-	rabbit	0.73	Wb, IF
GeneTex	GTX114073	43677	AB_10620339	polyclonal	-	rabbit	1.78	Wb
MilliporeSigma	G4171	0000181205	AB_1078958	polyclonal	-	rabbit	1.00	Wb
Proteintech	20622-1-AP	00013380	AB_10696317	polyclonal	-	rabbit	0.45	IF
Proteintech	27972-1-AP	00059099	AB_2881024	polyclonal	-	rabbit	0.60	Wb
Thermo Fisher Scientific	MA5-26589 *	YE3914194	AB_2724337	monoclonal	OTI1D12	mouse	1.00	Wb
Thermo Fisher Scientific	MA5-26591 *	YE3914195	AB_2724339	monoclonal	OTI4G4	mouse	1.00	Wb
Thermo Fisher Scientific	MA5-32591 **	YE3913381A	AB_2809868	recombinant mono	JM10-76	rabbit	1.00	Wb
Thermo Fisher Scientific	MA5-35236 **	YE3913567	AB_2849139	recombinant mono	ARC0500	rabbit	0.40	Wb
Thermo Fisher Scientific	MA5-38382 **	YE3914005	AB_2898296	recombinant mono	4H4	rabbit	0.40	Wb
Thermo Fisher Scientific	MA5-52914 **	ZJ4492612	AB_3249389	recombinant mono	23GB5775	rabbit	NA	Wb

Wb = Western blot; IF = Immunofluorescence; IP = Immunoprecipitation.

** = Recombinant antibody,

* = Monoclonal antibody, NA = Not available.

**Table 3.** Table of secondary antibodies used.

Company	Secondary antibody	Catalog number	RRID (Antibody registry)	Clonality	Concentration (μg/μL)	Working concentration (μg/mL)
Thermo Fisher Scientific	HRP-Goat Anti-Rabbit Antibody (H+L)	65-6120	AB_2533967	polyclonal	1.0	0.2
Thermo Fisher Scientific	HRP-Goat Anti-Mouse Antibody (H+L)	62-6520	AB_2533947	polyclonal	1.5	0.75
Cell Signaling Technology	Protein A, HRP conjugate	12291	NA	polyclonal	0.125	0.5
Thermo Fisher Scientific	Alexa Fluor™ 555-Goat anti-Rabbit IgG (H+L)	A-21429	AB_2535850	polyclonal	2.0	0.5
Thermo Fisher Scientific	Alexa Fluor™ 555-Goat anti-Mouse IgG (H+L)	A-21424	AB_141780	polyclonal	2.0	0.5

**Table 4.** Illustrations to assess antibody performance in all western blot, immunoprecipitation and immunofluorescence.

Western blot	Immunoprecipitation	Immunofluorescence
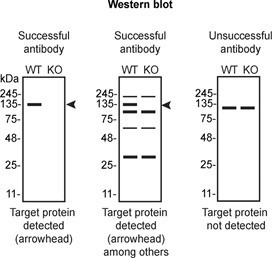	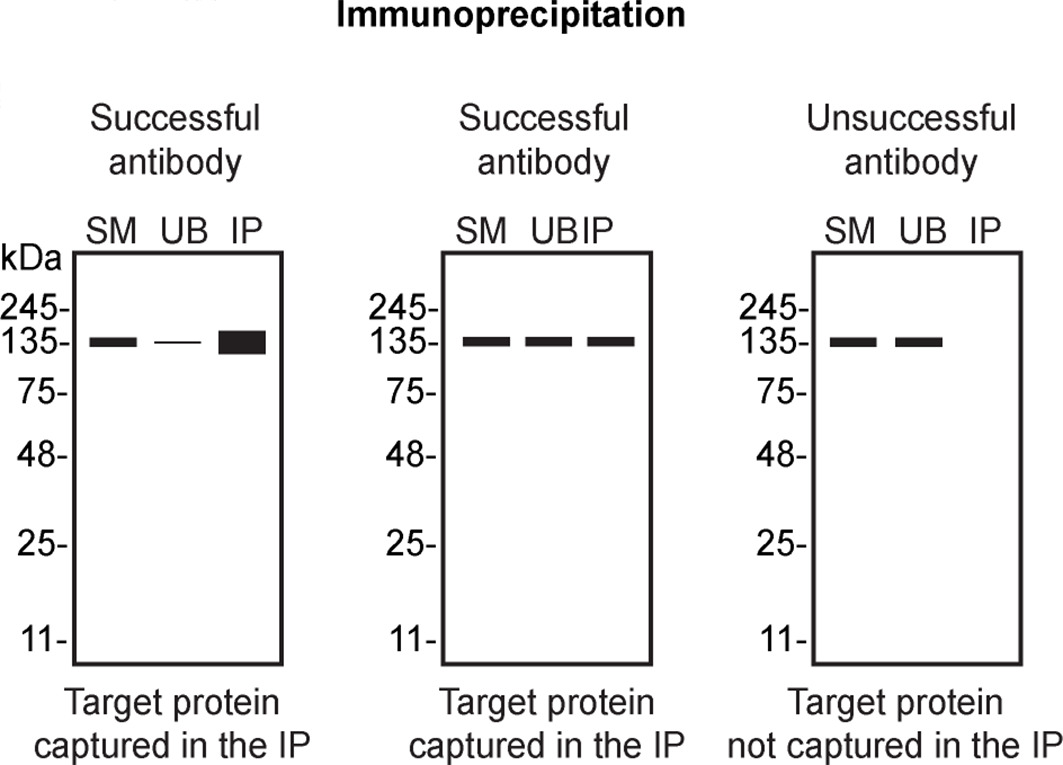	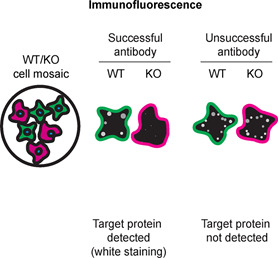

This table has been reproduced with permission from Ayoubi et al., Elife, 2023. ^7^

## Results and discussion

Our standard protocol involves comparing readouts from wild type (WT) and KO cells. ^5, 6^ The first step was to identify a cell line(s) that expresses sufficient levels of a given protein to generate a measurable signal using antibodies. To this end, we examined the DepMap (Cancer Dependency Map Portal, RRID:SCR_017655) transcriptomics database to identify all cell lines that express the target at levels greater than 2.5 log _2_ (transcripts per million “TPM” + 1), which we have found to be a suitable cut-off. ^7^ The HAP1 expresses the *GBA1* transcript at 3.5 log _2_ TPM+1. A *GBA1* KO HAP1 cell line was obtained from Horizon Discovery ( Table 1). Moreover, as seen on DepMap, the HAP1 does not carry mutations in the *GBA1* that could affect antibody–epitope binding.

**Figure 1.**
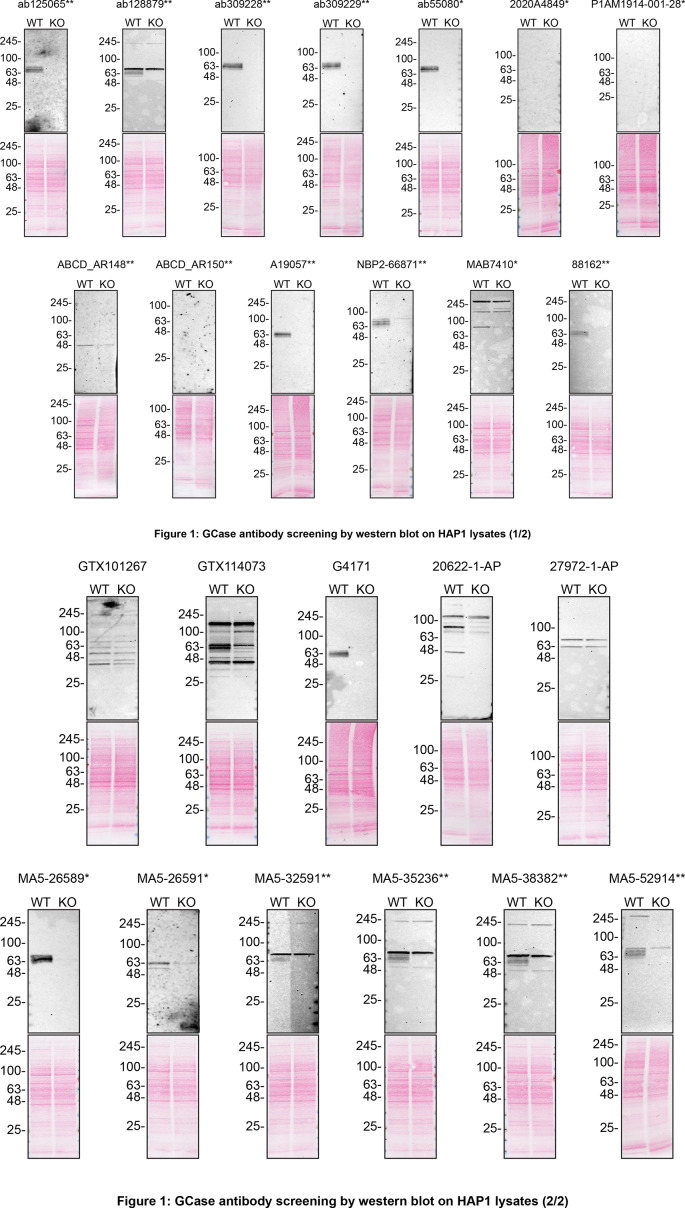
GCase antibody screening by western blot. Lysates of HAP1 WT and *GBA1* KO were prepared, and 30 μg of protein were processed for western blot with the indicated GCase antibodies. The Ponceau stained transfers of each blot are presented to show equal loading of WT and KO lysates and protein transfer efficiency from the acrylamide gels to the nitrocellulose membrane. Antibody dilutions were chosen according to the recommendations of the antibody supplier. Antibody dilutions used: ab125065** at 1/100, ab128879** at 1/1000, ab309228** at 1/200, ab309229** at 1/200, ab55080* at 1/500, 2020A4849* at 1/1000, P1AM1914-001-28* at 1/1000, ABCD_AR148** 1/50 (1 μg/mL), ABCD_AR150** at 1/20, A19057** at 1/1000, NBP2-66871** at 1/500, MAB7410* at 1/500, 88162** at 1/100, GTX101267 at 1/100, GTX114073 at 1/1000, G4171 at 1/1000, 20622-1-AP at 1/100, 27972-1-AP at 1/1000, MA5-26589* at 1/100, MA5-26591* at 1/100, MA5-32591** at 1/1000, MA5-35236** at 1/1000, MA5-38382** at 1/1000, and MA5-52914** at 1/1000. Predicted band size: 59.7 kDa. **= recombinant antibody, *= monoclonal antibody.

**Figure 2.**
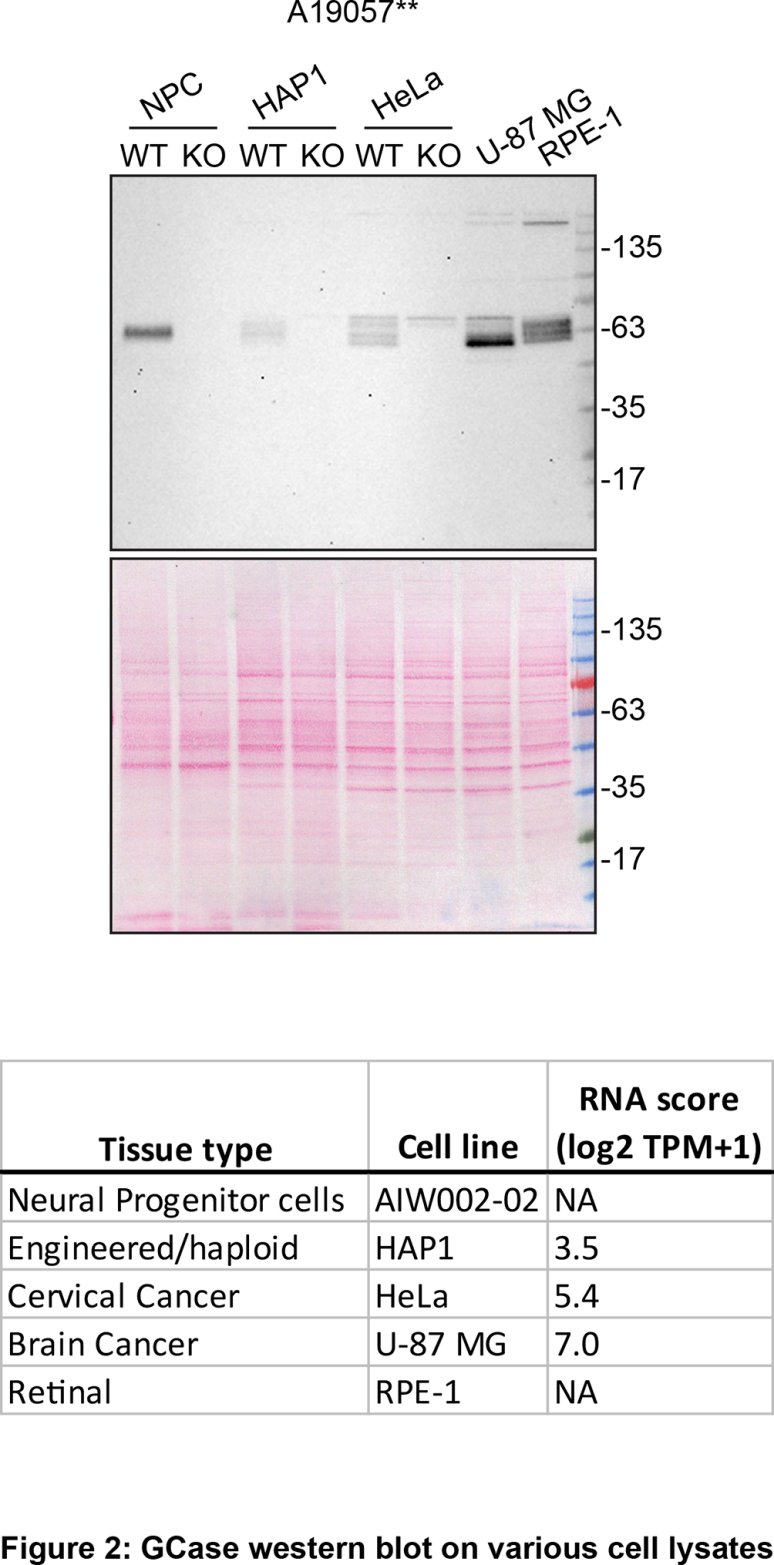
GCase western blot on various cell lysates. Lysates were prepared from WT and *GBA1* KOs in HAP1, neural progenitor cells and HeLa as well as WT U-87 MG and RPE-1. 30 μg of protein were processed for western blot with anti-GCase A19057** diluted at 1/1000. Predicted band size: 59.7 kDa. **= recombinant antibody.

To screen all twenty-four by western blot, WT and *GBA1* KO protein lysates were ran on SDS-PAGE, transferred onto nitrocellulose membranes, and then probed with twenty-four GCase antibodies in parallel ( Figure 1). HeLa expresses the *GBA1* transcript at 5.4 log _2_ TPM+1, and a HeLa *GBA1* KO is commercially available at Abcam. Protein expression of GCase was evaluated by western blot in the two cell types HAP1 and HeLa in addition to WT U-87 MG and RPE-1 ( Figure 2).

**Figure 3.**
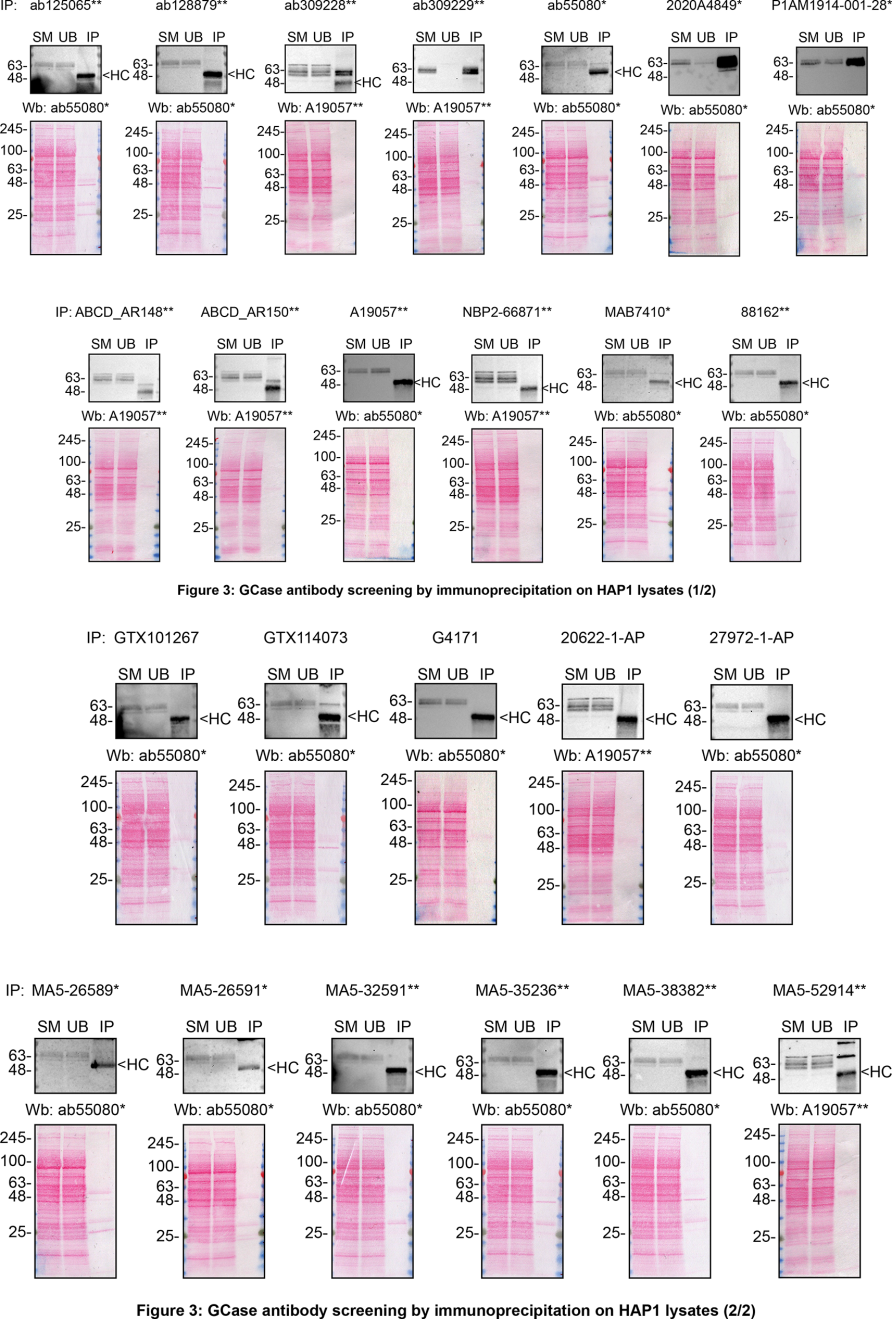
GCase antibody screening by immunoprecipitation on HAP1 lysates. HAP1 lysates were prepared, and immunoprecipitation was performed using 1 mg of lysate and 2.0 μg of the indicated GCase antibodies pre-coupled to Dynabeads protein A or protein G. Samples were washed and processed for western blot with the anti-GCase ab55080* diluted at 1/500 or A19057** at 1/1000. The Ponceau stained transfers of each blot are shown. SM=4% starting material; UB=4% unbound fraction; IP=immunoprecipitate, HC= antibody heavy chain. **= recombinant antibody, *= monoclonal antibody.

We then assessed the capability of all twenty-four antibodies to capture GCase from HAP1 protein extracts using immunoprecipitation techniques, followed by western blot analysis. For the immunoblot step, a specific GCase antibody identified previously (refer to Figure 1) was selected. Equal amounts of the starting material (SM) and the unbound fractions (UB), as well as the whole immunoprecipitate (IP) eluates were separated by SDS-PAGE ( Figure 3).

**Figure 4.**
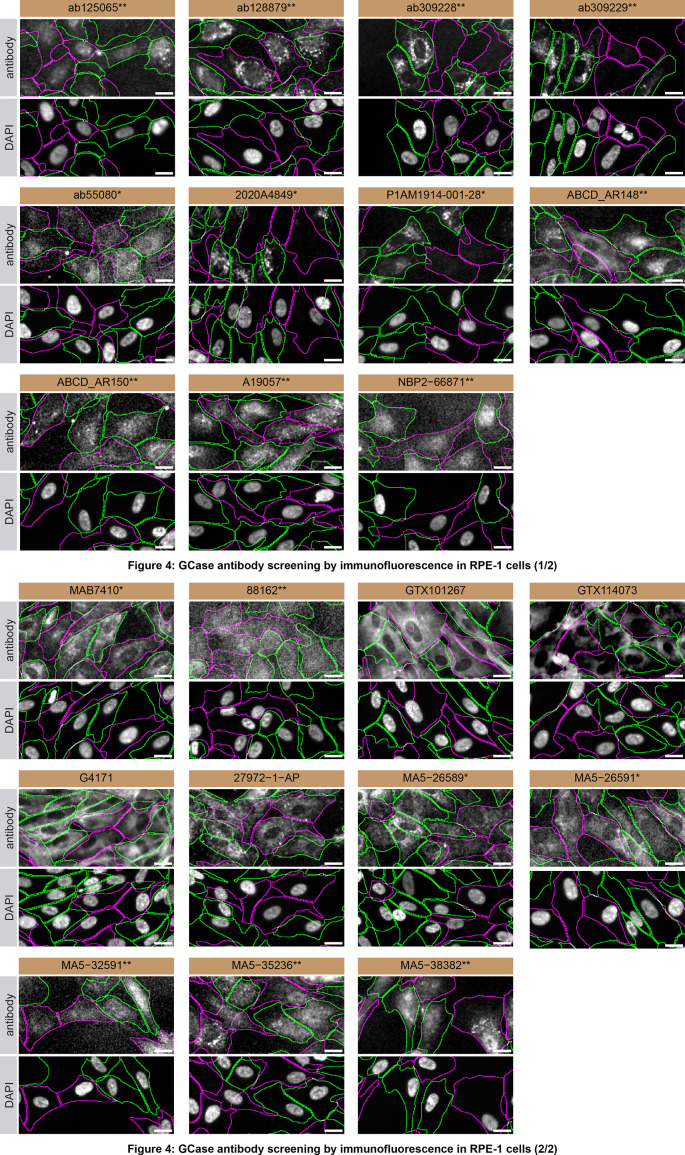
GCase antibody screening by immunofluorescence in RPE-1 cells. RPE-1 WT and *GBA1* KO cells were labelled with a green or a far-red fluorescent dye, respectively. WT and KO cells were mixed and plated to a 1:1 ratio on coverslips. Cells were stained with the indicated GCase antibodies and with the corresponding Alexa-fluor 555 coupled secondary antibody including DAPI. Acquisition of the blue (nucleus-DAPI), green (WT), red (antibody staining) and far-red (KO) channels was performed. Representative images of the merged blue and red (grayscale) channels are shown. WT and KO cells are outlined with green and magenta dashed line, respectively. When an antibody was recommended for immunofluorescence by the supplier, we tested it at the recommended dilution. The rest of the antibodies were tested at 1 and 2 μg/ml, and the final concentration was selected based on the detection range of the microscope used and a quantitative analysis not shown here. Antibody dilutions used: ab125065** at 1/50, ab128879** at 1/200, ab309228** at 1/500, ab309229** at 1/500, ab55080* at 1/1000, 2020A4849* at 1/500, P1AM1914-001-28* at 1/500, ABCD_AR148** 1/100, ABCD_AR150** at 1/100, A19057** at 1/500, NBP2-66871** at 1/1000, MAB7410* at 1/500, 88162** at 1/500, GTX101267 at 1/100, GTX114073 at 1/700, G4171 at 1/1000, 27972-1-AP at 1/300, MA5-26589* at 1/1000, MA5-26591* at 1/1000, MA5-32591** at 1/1000, MA5-35236** at 1/400, and MA5-38382** at 1/200. Bars = 10 μm. **= recombinant antibody, *= monoclonal antibody.

Based on the assessment of GCase expression in Figure 2, the highest expressing RPE-1 cells were selected for antibody screening in immunofluorescence. A KO cell line was generated by an academic partner in RPE-1 using CRISPR/Cas9. For immunofluorescence, twenty-two antibodies were screened using a mosaic strategy. First, RPE-1 WT and *GBA1* KO cells were labelled with different fluorescent dyes in order to distinguish the two cell lines, and the GCase antibodies were evaluated. Both WT and KO lines imaged in the same field of view to reduce staining, imaging and image analysis bias ( Figure 4). Quantification of immunofluorescence intensity in hundreds of WT and KO cells was performed for each antibody tested, and the images presented in Figure 4 are representative of this analysis. ^3^

This study confirms the utility of the mouse monoclonal antibodies clones 1/17 and 1/23 for immunoprecipitation and immunofluorescence, ^8^ and identifies rabbit recombinant antibodies suitable across multiple applications. The availability of both mouse- and rabbit-specific GCase antibodies enables multiplexing experiments and supports the development of antibody pairs for applications such as ELISA assays.

In conclusion, we have screened twenty-four GCase commercial antibodies by western blot, immunoprecipitation, and immunofluorescence by comparing the signal produced by the antibodies in human HAP1 and RPE-1 WT and *GBA1* KO cells. To assist users in interpreting antibody performanyce, Table 4 outlines various scenarios in which antibodies may perform in all three applications. ^7^ Several high-quality and renewable antibodies that successfully detect GCase were identified in all applications. Researchers who wish to study GCase in a different species are encouraged to select high-quality antibodies, based on the results of this study, and investigate the predicted species reactivity of the manufacturer before extending their research.

## Limitations

Inherent limitations are associated with the antibody characterization platform used in this study. Firstly, the YCharOS project focuses on renewable (recombinant and monoclonal) antibodies and does not test all commercially available GCase antibodies. YCharOS partners provide approximately 80% of all renewable antibodies, but some top-cited polyclonal antibodies may not be available through these partners. We encourage readers to consult vendor documentation to identify the specific antigen each antibody is raised against, where such information is available.

Secondly, the YCharOS effort employs a non-biased approach that is agnostic to the protein for which antibodies have been characterized. The aim is to provide objective data on antibody performance without preconceived notions about how antibodies should perform or the molecular weight that should be observed in western blot. As the authors are not experts in GCase, only a brief overview of the protein’s function and its relevance in disease is provided. GCase experts are invited to analyze and interpret observed banding patterns in western blots and subcellular localization in immunofluorescence.

Thirdly, YCharOS experiments are not performed in replicates primarily due to the use of multiple antibodies targeting various epitopes. Once a specific antibody is identified, it validates the protein expression of the intended target in the selected cell line, confirms the lack of protein expression in the KO cell line and supports conclusions regarding the specificity of the other antibodies. All experiments are performed using master mixes, and meticulous attention is paid to sample preparation and experimental execution. In IF, the use of two different concentrations serves to evaluate antibody specificity and can aid in assessing assay reliability. In instances where antibodies yield no signal, a repeat experiment is conducted following titration. Additionally, our independent data is performed subsequently to the antibody manufacturers internal validation process, therefore making our characterization process a repeat.

Lastly, as comprehensive and standardized procedures are respected, any conclusions remain confined to the experimental conditions and cell line used for this study. The use of a single cell type for evaluating antibody performance poses as a limitation, as factors such as target protein abundance significantly impact results. Additionally, the use of cancer cell lines containing gene mutations poses a potential challenge, as these mutations may be within the epitope coding sequence or other regions of the gene responsible for the intended target. Such alterations can impact the binding affinity of antibodies. This represents an inherent limitation of any approach that employs cancer cell lines.

## Method

The standardized protocols used to carry out this KO cell line-based antibody characterization platform was established and approved by a collaborative group of academics, industry researchers and antibody manufacturers. The detailed materials and step-by-step protocols used to characterize antibodies in western blot, immunoprecipitation and immunofluorescence are openly available on Protocols.io ( protocols.io/view/a-consensus-platform-for-antibody-characterization ). ^3^ Brief descriptions of the experimental setup used to carry out this study can be found below.

### Cell lines and antibodies

The cell lines, primary and secondary antibodies used in this study are listed in Tables 1, 2, and 3, respectively. To ensure consistency with manufacturer recommendations and account for proprietary formulations (where antibody concentrations are not disclosed), antibody usage is reported as dilution ratios rather than absolute concentrations. To facilitate proper citation and unambiguous identification, all cell lines and antibodies are referenced with their corresponding Research Resource Identifiers (RRIDs). ^9, 10^ HAP1 KO clone corresponding to the *GBA1* was generated with low passage cells at Horizon Discovery. Guide RNA (CCCATCCAGGCTAATCACAC) were used to induce a 23bp deletion in the exon 3 of *GBA1* gene. All cell lines used in this study were regularly tested for mycoplasma contamination and were confirmed to be mycoplasma-free.

### Antibody screening by western blot

HAP1 WT and *GBA1* KO cells were collected in RIPA buffer (25mM Tris-HCl pH 7.6, 150mM NaCl, 1% NP-40, 1% sodium deoxycholate, 0.1% SDS) (Thermo Fisher Scientific, cat. number 89901) supplemented with 1x protease inhibitor cocktail mix (MilliporeSigma, cat. number P8340). Lysates were sonicated briefly and incubated 30 min on ice. Lysates were spun at ~110,000 *x g* for 15 min at 4°C and equal protein aliquots of the supernatants were analyzed by SDS-PAGE and western blot. BLUelf prestained protein ladder (GeneDireX, cat. number PM008-0500) was used.

Western blots were performed with precast midi 4-20% Tris-Glycine polyacrylamide gels (Thermo Fisher Scientific, cat. number WXP42012BOX) ran with Tris/Glycine/SDS buffer (Bio-Rad, cat. number 1610772), loaded in Laemmli loading sample buffer (Thermo Fisher Scientific, cat. number AAJ61337AD) and transferred on nitrocellulose membranes. Proteins on the blots were visualized with Ponceau S staining (Thermo Fisher Scientific, cat. number BP103-10) which is scanned to show together with individual western blot. Blots were blocked with 5% milk for 1 hr, and antibodies were incubated O/N at 4°C with 5% milk in TBS with 0,1% Tween 20 (TBST) (Cell Signalling Technology, cat. number 9997). Following three washes with TBST, the peroxidase conjugated secondary antibody was incubated at a dilution of ~0.2 μg/ml in TBST with 5% milk for 1 hr at room temperature followed by three washes with TBST. Membranes were incubated with Pierce ECL (Thermo Fisher Scientific, cat. number 32106) Or Clarity Western ECL Substrate (Bio-Rad, cat. number 1705061) prior to detection with the iBright™ CL1500 Imaging System (Thermo Fisher Scientific, cat. number A44240).

### Antibody screening by immunoprecipitation

Antibody-bead conjugates were prepared by adding 2 μg to 500 μl of Pierce IP Lysis Buffer from Thermo Fisher Scientific (cat. number 87788) in a microcentrifuge tube, together with 30 μl of Dynabeads protein A- (for rabbit antibodies) or protein G- (for mouse antibodies) (Thermo Fisher Scientific, cat. number 10002D and 10004D, respectively). Anti-GCase MA5-52914** was at an unknown concentration and thus 10 μl were tested in IP. All tubes were rocked for ~1 h at 4°C followed by two washes to remove unbound antibodies. HAP1 WT were collected in Pierce IP buffer (25 mM Tris-HCl pH 7.4, 150 mM NaCl, 1 mM EDTA, 1% NP-40 and 5% glycerol) supplemented with protease inhibitor. Lysates were rocked 30 min at 4°C and spun at 110,000 *x g* for 15 min at 4°C. 0.5 ml aliquots at 1 mg/ml of lysate were incubated with an antibody-bead conjugate for ~1 h at 4°C. The unbound fractions were collected, and beads were subsequently washed three times with 1.0 ml of IP buffer and processed for SDS-PAGE and western blot on precast midi 4-20% Tris-Glycine polyacrylamide gels. Protein A:HRP was used as a secondary detection system at a concentration of 0.5 μg/ml.

### Antibody screening by immunofluorescence

RPE-1 WT and *GBA1* KO cells were labelled with a green and a far-red fluorescence dye, respectively (Thermo Fisher Scientific, cat. number C2925 and C34565). The nuclei were labelled with DAPI (Thermo Fisher Scientific, cat. Number D3571) fluorescent stain. WT and KO cells were plated on 96-well plate with optically clear flat-bottom (Perkin Elmer, cat. number 6055300) as a mosaic and incubated for 24 hrs in a cell culture incubator at 37 ^o^C, 5% CO _2_. Cells were fixed in 4% paraformaldehyde (PFA) (VWR, cat. number 100503-917) in phosphate buffered saline (PBS) (Wisent, cat. number 311-010-CL). Cells were permeabilized in PBS with 0,1% Triton X-100 (Thermo Fisher Scientific, cat. number BP151-500) for 10 min at room temperature and blocked with PBS with 5% BSA, 5% goat serum (Gibco, cat. number 16210-064) and 0.01% Triton X-100 for 30 min at room temperature. Cells were incubated with IF buffer (PBS, 5% BSA, 0,01% Triton X-100) containing the primary GCase antibodies overnight at 4°C. Cells were then washed 3 × 10 min with IF buffer and incubated with corresponding Alexa Fluor 555-conjugated secondary antibodies in IF buffer at a dilution of 1.0 μg/ml for 1 hr at room temperature with DAPI. Cells were washed 3 × 10 min with IF buffer and once with PBS.

Images were acquired on an ImageXpress micro confocal high-content microscopy system (Molecular Devices), using a 20x NA 0.95 water immersion objective and scientific CMOS cameras, equipped with 395, 475, 555 and 635 nm solid state LED lights (lumencor Aura III light engine) and bandpass filters to excite DAPI, Cellmask Green, Alexa-555 and Cellmask Red, respectively. Images had pixel sizes of 0.68 x 0.68 microns, and a z-interval of 4 microns. For analysis and visualization, shading correction (shade only) was carried out for all images. Then, maximum intensity projections were generated using 3 z-slices. Segmentation was carried out separately on maximum intensity projections of Cellmask channels using CellPose 1.0, and masks were used to generate outlines and for intensity quantification. ^11^ Figures were assembled with Adobe Illustrator.

## Data Availability

Zenodo: Dataset for the GCase antibody screening study ( https://doi.org/10.5281/zenodo.17693514) Data are available under the terms of the Creative Commons Attribution 4.0 International license (CC-BY 4.0).

## References

[1] Parlar SC Grenn FP Kim JJ : Classification of GBA1 Variants in Parkinson’s Disease: The GBA1-PD Browser. *Mov. Disord.* 2023;38(3):489–495. 10.1002/mds.29314 36598340 PMC10033371

[2] Orvisky E Park JK Parker A : The identification of eight novel glucocerebrosidase (GBA) mutations in patients with Gaucher disease. *Hum. Mutat.* 2002;19(4):458–459. 10.1002/humu.9024 11933202

[3] Ayoubi R Ryan J Gonzalez Bolivar S : A consensus platform for antibody characterization. *Nat. Protoc.* 2024. 10.1038/s41596-024-01095-8 PMC13054593 39690206

[4] Biddle MS Virk HS : YCharOS open antibody characterisation data: Lessons learned and progress made. *F1000Res.* 2023;12:1344. 10.12688/f1000research.141719.1 37854875 PMC10579855

[5] Laflamme C McKeever PM Kumar R : Implementation of an antibody characterization procedure and application to the major ALS/FTD disease gene C9ORF72. *elife.* 2019;8. 10.7554/eLife.48363 31612854 PMC6794092

[6] Alshafie W Fotouhi M Shlaifer I : Identification of highly specific antibodies for Serine/threonine-protein kinase TBK1 for use in immunoblot, immunoprecipitation and immunofluorescence. *F1000Res.* 2022;11:977. 10.12688/f1000research.124632.1 36415206 PMC9647147

[7] Ayoubi R Ryan J Biddle MS : Scaling of an antibody validation procedure enables quantification of antibody performance in major research applications. *elife.* 2023;12:12. 10.7554/eLife.91645.2 PMC10666931 37995198

[8] Jong T Gehrlein A Sidransky E : Characterization of Novel Human beta-glucocerebrosidase Antibodies for Parkinson’s Disease Research. *J. Parkinsons Dis.* 2024;14(1):65–78. 10.3233/JPD-230295 38251062 PMC10836542

[9] Bandrowski A Pairish M Eckmann P : The Antibody Registry: ten years of registering antibodies. *Nucleic Acids Res.* 2023;51(D1):D358–D367. 10.1093/nar/gkac927 36370112 PMC9825422

[10] Bairoch A : The Cellosaurus, a Cell-Line Knowledge Resource. *J. Biomol. Tech.* 2018;29(2):25–38. 10.7171/jbt.18-2902-002 29805321 PMC5945021

[11] Stringer C Wang T Michaelos M : Cellpose: a generalist algorithm for cellular segmentation. *Nat. Methods.* 2021;18(1):100–106. 10.1038/s41592-020-01018-x 33318659

