# Supplementary material for: A guide to selecting high-performing antibodies for GCase (UniProt ID: P04062) for use in western blot, immunoprecipitation, and immunofluorescence
Source: F1000Res. 2025 Dec 15;14:1400. [Version 1] doi: 10.12688/f1000research.174230.1 (PMC12800601; doi:10.12688/f1000research.174230.1)
Supplement: Peer review history

# Reviewer report: Reviewer response for version 1

**DOI:** 10.5256/f1000research.192114.r446553

**Published:** 2026-01-13

**Authors:** Karen Bowman

**Affiliations:** 1 University of Leicester, Leicester, England, UK

The study involved characterisation of twenty-four commercial antibodies, from several different companies, for the human glucocerebrosidase (GCase) protein for use in western blot, immunoprecipitation, and immunofluorescence. The study formed part of the wider YCharOS collaboration to characterise commercial antibodies for human proteins using standardised protocols with the intention of improving antibody reproducibility issues. As a Data Note, it allows the direct comparison of the performance of commercially available antibodies to the GCase protein to aid scientists in choosing the most appropriate antibody for their studies. For example, in understanding how disease-related mutations affect the protein’s biochemical and cellular characteristics. This comparison would be of particular use in the study of Parkinson’s and Gaucher’s disease.

 A committee of industry and academic representatives have endorsed the platform used, and the protocols are consistent with those in general use. The platform consisted of identification of human cell lines suitable for antibody characterisation studies i.e., with adequate levels of the GCase protein to generate a measurable signal. The final step was a series of antibody characterisation procedures, limited to the most common research uses of antibodies. They found that the commercially available HAP1 cell line expresses *GBA1* transcript at a level above the average range for cancer cells analysed, and therefore, was a logical choice for their study. A further three cell lines (HeLa, WT U-87 MG and RPE-1) and neural progenitor cells, which also express *GBA1* transcript at high levels, were evaluated by western blot to determine GCase expression levels. Knockdown of the target gene was used to provide appropriate negative control cells for each cell line ( *GBA1* KO). Knockout cells were obtained from either commercial sources or, in the case of RPE-1 *GBA1* KO, from an academic partner utilising CRISPR/Cas9 technology. The antibodies were evaluated by western blot and immunoprecipitation in HAP1 wild type and HAP1 *GBA1* KO cells. However, the immunofluorescence studies were conducted in RPE-1 and RPE-1 *GBA1* KO cells as they exhibited the highest levels of GCase expression in a western blot comparison assay. The interpretation of the results is left to the reader, with the study providing unbiased guidance. However, it gave some indication of the optimum dilutions of commercial antibodies. Limitations of the study are clearly stated. The study indicates variability in the specificity of the antibodies within and between assays. It highlights the importance of selecting the most appropriate anti-GCase antibody for each specific assay. At face value, the use of the RPE-1 *GBA1* KO cell line to compare binding of the antibodies in the presence and absence of GCase protein is acceptible, but more information and a reference and/or a link for the RPE-1 *GBA1* KO cells would help with interpretation of the results.

Are sufficient details of methods and materials provided to allow replication by others?

Yes

Is the rationale for creating the dataset(s) clearly described?

Yes

Are the datasets clearly presented in a useable and accessible format?

Yes

Are the protocols appropriate and is the work technically sound?

Yes

Reviewer Expertise:

Drug development, cell biology

I confirm that I have read this submission and believe that I have an appropriate level of expertise to confirm that it is of an acceptable scientific standard.

# Reviewer report: Reviewer response for version 1

**DOI:** 10.5256/f1000research.192114.r442397

**Published:** 2026-01-10

**Authors:** Deborah Moshinsky

**Affiliations:** 1 Institute for Protein Innovation, Boston, Massachusetts, USA

The authors tested 24 commercially available antibodies to GCase for their ability to bind to antigen in Western Blotting, Immunoprecipitation, and Immunofluorescence.  Cell lines expressing the target at high levels were used and comparisons were made to knockouts.  Renewable antibodies that detect GCase were identified in all applications.  The authors present general guidance in data interpretation and state sufficient reasons for not engaging in full data interpretation.  They also clearly specify limitations of their studies.  Overall, this study should provide a great resource for those seeking validated antibodies for GCase in the applications tested using standardized and clearly defined protocols.  The authors are encouraged to perform a spell check, as an error was found (i.e. "performanyce").

Are sufficient details of methods and materials provided to allow replication by others?

Yes

Is the rationale for creating the dataset(s) clearly described?

Yes

Are the datasets clearly presented in a useable and accessible format?

Yes

Are the protocols appropriate and is the work technically sound?

Yes

Reviewer Expertise:

Antibody Characterization and Validation

I confirm that I have read this submission and believe that I have an appropriate level of expertise to confirm that it is of an acceptable scientific standard.
